# Environmental Drivers of Weed Floristic Diversity in Two Contrasting Sugarcane Agroecosystems

**DOI:** 10.3390/plants15121825

**Published:** 2026-06-12

**Authors:** Mohamed Abdelazeem Mousa, Ahmed K. Osman, Mashail N. Alzain, Oqba Basal, Mohamed Kamel, Sabah A. Hammad, Naglaa Loutfy, Mohamed O. Badry

**Affiliations:** 1 Department of Applied Plant Biology, Faculty of Agricultural and Food Sciences and Environmental Management, University of Debrecen, 4032 Debrecen, Hungary; mohamed.abdelazeem@sci.svu.edu.eg; 2 Department of Botany & Microbiology, Faculty of Science, Qena University, Qena 83523, Egypt; ahmosman2000@yahoo.com (A.K.O.); mohamedkamelahmed@yahoo.com (M.K.); sabah.hammad@sci.svu.edu.eg (S.A.H.); naglaa.hasssn@sci.svu.edu.eg (N.L.); 3 Department of Biology, College of Science, Princess Nourah bint Abdulrahman University, P.O. Box 84428, Riyadh 11761, Saudi Arabia; mnalzain@pnu.edu.sa

**Keywords:** floristic composition, Nile lands, reclaimed lands, seasonality, sugarcane, TWINSPAN, Simpson’s Index, Shannon-Wiener Index, species evenness, edaphic factors

## Abstract

Sugarcane is a high-value crop in Egypt, yet weed communities in the understudied Upper Egypt region have not been systematically characterized. This study provides a comprehensive analysis of weed floristic composition, phytogeographical affinities, and the edaphic and canopy light factors governing vegetation structure across contrasting Nile Valley clay and reclaimed desert lands in Qena Governorate. Fourteen stands were surveyed during the 2024/2025 sugarcane growing season, recording 110 species from 33 families (68 annuals and 42 perennials), which were dominated by Poaceae, Asteraceae, Fabaceae, Euphorbiaceae, and Amaranthaceae (54.6% of the flora recorded). Therophytes were the most abundant life form (60.9%), and 51.8% of species belonged to Neotropical, Palaeotropical, Cosmopolitan, and Pantropical chorotypes. Diversity indices showed high and balanced species diversity, with no dominance by any single species. Seasonal variation showed that species richness peaked in spring, decreased through summer and autumn, and correlated with light intensity under the canopy. TWINSPAN identified four vegetation groups, which were merged into three primary vegetation groups (A, B, and C) via DCA and CCA ordinations and linked to microhabitats shaped by elevation and soil physicochemical properties. CCA revealed that Group C (stands in the Nile Riverbank lands) had the highest diversity, which was associated with organic matter, clay, and field capacity. In contrast, Group A (stands of reclaimed desert land) had low richness linked to high levels of Total Dissolved Solids (TDS), Electrical Conductivity (EC), Na, K, Mg, CaCO_3_, and sandy soils. Group B (stands of Nile clay lands) was an intermediate transitional community between groups A and C. These findings establish edaphic factors as the primary determinant of weed community structure, with salinity as the critical constraint in reclaimed lands and seasonal light variation as a secondary diversity filter.

## 1. Introduction

Agriculture is one of the most important sectors in Egypt’s economy. Egypt’s agriculture sector faces numerous challenges in meeting the growing domestic food demand and reducing poverty. According to El-Khalifa and Zahran [1], Egypt’s agricultural area was estimated at 3.86 million hectares, approximately 4% of the country’s total land area. This area was mainly located on the banks of the Nile and the canal. Rapid large-scale agricultural expansion has occurred through land reclamation to combat desertification and meet the country’s growing demand.

In Egypt, land reclamation uses underground water resources or extends water canals from current agricultural land into the desert to establish rural settlements and turn desert areas into agricultural land. Certain crops can be grown rather well in areas of these regions with loamy soil [2]. In Upper Egypt, sugarcane has recently been planted alongside wheat, maize, and barley on cultivated land.

Sugarcane (*Saccharum officinarum* L.) is a rhizomatous geophyte that grows in tropical locations that are seasonally dry. Approximately 80% of the world’s sugar is produced from sugarcane, making it one of the most significant cash crops [3]. About 37.8% of the 2.458 million tons of sugar harvested in Egypt in 2019 originated from sugarcane [4]. Nowadays, sugarcane is grown along the Nile River in the Governorates of Menya, Sohag, Qena, Luxor, and Aswan in Upper Egypt [4]. According to Abdel Daiem et al. [5] sugarcane was grown on 104.2 thousand hectares of land in Egypt in 2021, with an average yield of 91.7 tons per hectare.

Sugarcane cultivation in Egypt faces significant challenges, primarily due to the crop’s need for extensive irrigation in an arid region, where competing demands on the Nile’s water supplies are rapidly increasing [6]. According to Farag et al. [7], the growth of sugarcane requires a significant amount of irrigation water, roughly 29,518 m^3^/hectare. Furthermore, yields are reduced by severe degradation caused by extended monocropping and soil salinity. Additionally, rising production costs, heat stress caused by climate change, and competition from associated weeds make sugarcane production less economically viable for small farmers [8].

Weeds are highly detrimental to sugarcane production and have been reported to cause yield losses of 20–90% in various countries [9,10]. Diverse weed species that compete for nutrients, water, and light are common in sugarcane plantations, which affects sugarcane yield. Common associated weed species include *Cynodon dactylon* (L.) Pers. (Bermuda grass), *Sorghum halepense* (L.) Pers. (Johnson grass), and *Cyperus rotundus* L. (purple nutsedge), which are highly persistent and difficult to control [11]. These weeds not only reduce sugarcane yields but also serve as hosts for pests and diseases, posing further agricultural challenges. However, weeds are crucial to ecosystem functions, food webs, nutrient cycling, plant community structure, and the availability of animal habitats [12]. A previous study by Chen et al. [13] demonstrated that weeds can be utilized to enhance species diversity within an ecosystem and maintain soil fertility. Moreover, Fried et al. [14] reported that arable weed species are essential for maintaining biological diversity, especially as they serve as the main food source for insects and birds living in farmland.

Furthermore, a variety of variables, such as crop type, season, human activities, and soil properties, influence weed communities, act simultaneously and make it difficult to assess the relative significance of the numerous elements that contribute to their development [15]. Studying floristic composition provides information about species distribution, ecosystem health, and ecological interactions, and understanding them is essential for evaluating biodiversity [16]. Additionally, analyzing plant communities supports effective weed management by identifying aggressive or dominant native species and their competitive dynamics, enabling targeted control strategies [17].

In agroecosystems, weed assembly dynamics are jointly governed by temporal shifts in microclimate and spatial heterogeneity in edaphic traits [18]. Temporally, the rapid growth of the sugarcane canopy creates a progressive light attenuation gradient, heavily influencing seasonal weed germination and structural shifts from therophyte-dominated spring flushes to shade-tolerant or perennial cohorts in later stages [19]. Spatially, the stark contrast between the traditional, fertile alluvial soils of the Nile Valley and the newly reclaimed desert lands creates profound environmental gradients [20]. While old Nile Valley lands typically feature high clay contents, organic matter, and superior field capacities, reclaimed desert soils are frequently constrained by coarse sandy textures, high total dissolved solids (TDS), elevated electrical conductivity (EC), and excessive concentrations of soluble salts (Na^+^, K^+^, and Mg^2+^) [20,21].

Despite the agricultural importance of sugarcane in Upper Egypt, the ecological drivers structuring its associated weed communities remain poorly understood, particularly under the contrasting soil conditions of the Nile Valley and reclaimed lands. Understanding how abiotic and biotic factors interact to shape weed diversity is essential for predicting vegetation dynamics in these agroecosystems. We hypothesize that (i) Weed diversity in sugarcane fields is lower in reclaimed lands than in the Nile Valley due to harsher environmental conditions, and (ii) Weed diversity is highest in spring and declines where sugarcane canopy development exerts a stronger regulatory effect on weed communities and vegetation structure than soil properties when light becomes the primary limiting resource. Therefore, this study addressed the following research questions: (1) What is the floristic composition, taxonomic structure, and life form spectra of weed communities in sugarcane fields across Nile Valley clay versus reclaimed desert lands in Qena Governorate? (2) What are the phytogeographical affinities of the weed flora associated with these sugarcane agroecosystems? (3) How do weed diversity and richness differ between the two land types, and how does seasonal canopy development influence species richness across these systems? (4) What distinct phytosociological community groups characterize these agroecosystems, and which edaphic variables drive their differentiation?

## 2. Materials and Methods

### 2.1. Study Area

**Figure 1:**
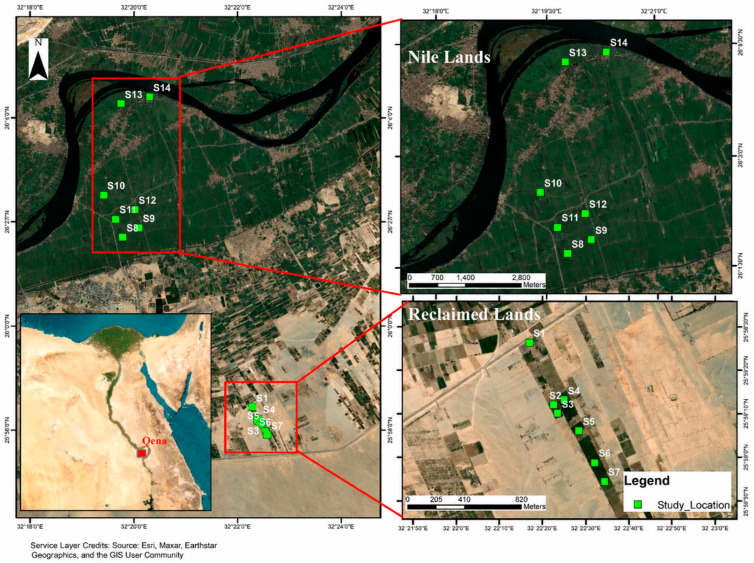
Location map of the study area at Qena Governorate, Upper Egypt (green rectangles), showing the sampling sites (S1–S14).

Field surveys were conducted from the beginning of sugarcane growth in May 2024 until harvesting in February 2025 at sugarcane fields in Nag Hamady, Qena Governorate, Egypt. This survey was conducted in two types of land (reclaimed desert lands and Nile basin lands). The study area extends to around 25°58′13.08″ N and 32°22′25.07″ E for reclaimed land, and 26°02′03″ N and 32°19′39″ E for the Nile basin lands (Figure 1).

The reclaimed lands in Upper Egypt lie at the boundaries between the desert and the Nile Valley and have been developed to increase agricultural crop production, relying on groundwater as the primary irrigation resource. The soil in these reclaimed areas is typically sandy, with poor water retention capacity, low inherent fertility, low organic matter content, a coarse texture, high calcium carbonate levels, and high salinity. On the other hand, the Nile Valley, the main area of cultivation in Egypt, has fertile soil and is irrigated with sweet water.

**Figure 2:**
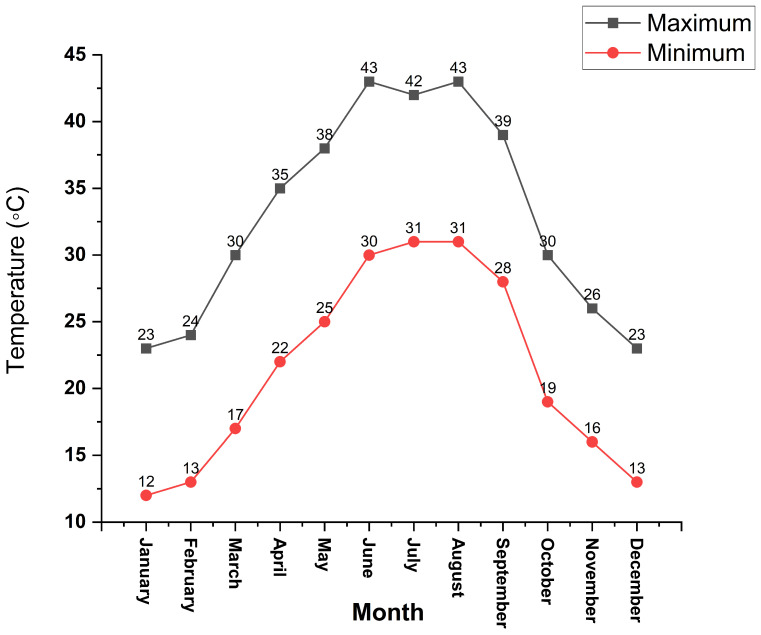
Monthly maximum and minimum air temperature (°C) in Qena Governorate throughout the year 2024.

Qena Governorate’s climate is subtropical desert, with hot, dry summers, mild winters, and wide daily temperature variations. This is due to its location in a high-pressure zone, which limits rainfall and allows intense solar radiation to penetrate. The highest temperature in summer was 43 °C in the summer months (June and August 2024), and the lowest temperature was 12 °C in the winter months (December 2024 and January 2025) (measured at meteorological station of Qena University at Qena City, Egypt; 26°12′00.0″ N, 32°45′00.0″ E, 96 m above sea level) (Figure 2).

### 2.2. Vegetation Sampling and Species Identification

**Figure 3:**
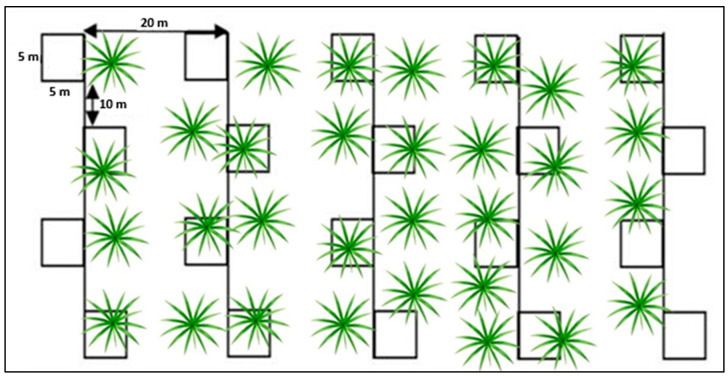
The sampling plot design for weed collection in sugarcane fields was generated in Adobe Illustrator CS6.

Four trips were made to the Sugarcane fields over the course of the study period, from May 2024 to February 2025, spanning four seasons (spring, summer, autumn, winter), until the harvesting of juicy sugarcane plants. We sampled 14 georeferenced vegetation stands (fields): seven in the reclaimed desert lands and seven in the Nile basin lands. Each field measured 100 m × 100 m. Within each field, the field survey was conducted using the method described by Kamal-Uddin et al. [22] and Takim and Amodu [23], where five parallel transects (each ran the full length of the field, 100 m) spaced at 20 m intervals were established. Along each transect, seven permanent quadrats (5 m × 5 m each) were placed at 10 m intervals, alternating between the left and right sides of the transect line. This design yielded 35 quadrats per field (5 transects × 7 quadrats per transect), and a total of 490 quadrats were sampled across the entire study (Figure 3).

Although classical vegetation ecology recommends different quadrat sizes for different growth forms [24], the standardized 5 × 5 m quadrat was retained for all growth forms in this study to account for the row spacing of the sugarcane crop (1.2–1.5 m), ensuring that each quadrat encompassed both cane rows and interrow spaces, thereby capturing the full microhabitat heterogeneity of the weed community; this method was chosen because of the low overall weed density and the need for consistent representation of row–interrow patterns across all growth forms [25].

During the study, wild weeds were physically collected from the sugarcane fields with the consent of the participating landowners/farmers. The study area is not a nature reserve, and collecting plant samples for study is permitted. Taxonomic identification was performed according to the available literature [26,27,28,29,30], and updated according to the Plants Of The World Online website [31] provided by the Royal Botanic Gardens, Kew. For each stand, we recorded the number of individuals per species. Life form spectra were determined using Raunkiaer’s classification system [32]. Each life form’s species number is represented as a percentage of the study area’s total species count. Phytogeographical chorotypes (floristic regions) for each recorded weed species were determined following the classification of White [33]. The collected weed specimens were dried, and vouchers were deposited in the Herbarium of the Faculty of Science, Qena University (QNA), Egypt. The voucher specimen ID numbers are presented in Table S1.

### 2.3. Soil Sampling and Analyses

Three soil samples were collected at 0–20 cm depth from randomly selected points within each stand. The three samples from the same stand were combined. After homogenizing the air-dried soil samples, the gravel was removed by passing them through a 2 mm screen. The percentages of sand, silt, and clay in the soil were determined using the pipette method [34]. The weight loss following ignition at 600 °C was used to calculate the total organic matter [35]. The physical and chemical properties of the soil, including soil moisture, texture, pH of soil extract, electrical conductivity (EC), total dissolved solids (TDS), and mineral content, were estimated. Both EC and TDS were determined using a conductivity meter (model 4520, JENWAY UK, Bibby Scientific Ltd., Dunmow, UK). pH was measured using a pH meter (model MW102, Milwaukee, WI, USA). Major cations and anions (K^+^, Ca^2+^, Mg^2+^, Na^+^, Cl^−^, SO_4_^2−^, NO_3_^−^) in the soil extracts were estimated as mg/g according to the method of Estefan et al. [36]. Soil alkalinity and field capacity were determined according to the method of Jackson [37].

### 2.4. Light Intensity Measurement Under the Sugarcane Canopy

A digital lux meter (Model LX1010B, Century Harvest Electronics Co., Ltd., Shenzhen, Guangdong, China) was used to measure canopy light penetration within the sugarcane field across the four seasons. The method described by Inman-Bamber [38] was used to quantify light intensity at the soil surface beneath the green canopy. In addition to quantifying light attenuation through sugarcane canopy and its effect on weed diversity, these measurements provided seasonal estimates of under-canopy illumination.

### 2.5. Composition of Weed Communities

To compare the alpha diversity of weed species between sugarcane fields in the Nile Valley and reclaimed lands, we applied three complementary indices: Simpson’s Index (D), which is sensitive to dominant species; the Shannon-Wiener Index (H′), which measures richness and rare species; and Evenness (E), which reflects the uniformity of species distribution. These indices were calculated for each plant community by determining the presence of species per stand using the Paleontological Statistics Software Package (PAST5) according to Hammer and Harper [39]. Comparing these indices allows us to assess whether diversity differences, if present, between sugarcane fields in the Nile Valley and reclaimed lands are driven by changes in dominant species or by shifts in rare species composition.

The floristic data matrix for the 14 studied stands was classified using Two-Way Indicator Species Analysis (TWINSPAN), selected for its ability to simultaneously classify stands and species into discrete groups based on indicator taxa, which revealed the hierarchical structure of weed communities across contrasting agroecological conditions. Detrended Correspondence Analysis (DCA) was applied to visualize floristic relationships among stands along continuous environmental gradients without assuming linear species responses, effectively removing the arch effect inherent in standard correspondence analysis, making it suitable for analyzing long floristic gradients [40]. Canonical Correspondence Analysis (CCA) was subsequently applied to directly relate species composition to measured soil variables, quantifying the relative contribution of each edaphic factor to community differentiation [41]. Together, these three techniques provided a complementary analytical framework: TWINSPAN defined discrete community groups, DCA revealed underlying floristic gradients, and CCA linked those gradients explicitly to soil properties. The Kolmogorov–Smirnov and Levene’s tests were used to assess normality and homogeneity before parametric testing, with a significance level of 0.05 applied throughout. A Student’s *t*-test was used to examine differences in species richness and diversity indices between the two land types. At the same time, a one-way ANOVA with Tukey’s HSD post hoc test revealed significant differences in soil variables among groups. All analyses were performed in SPSS 23.0 for Windows (SPSS Inc., Chicago, IL, USA).

## 3. Results

### 3.1. Floristic Diversity and Taxonomic Structure

Sugarcane fields across the Nile Valley clay and reclaimed desert lands in Qena Governorate harbored a taxonomically rich and ecologically diverse weed flora, comprising 110 flowering plant species across 88 genera and 33 families, with compositional identity differing significantly between the two contrasting land types (Table A1). Dicots were represented by 28 families and 96 taxa (84.27%), while monocots were represented by five families and 14 taxa (12.73%). The most species-rich families were Poaceae (17.27%), Asteraceae (10.91%), Fabaceae (10%), and Euphorbiaceae (9.09%). In comparison, Amaranthaceae was represented by six taxa (7.27%), and Convolvulaceae and Malvaceae by five species each (4.55%). These seven families accounted for 63.64% of the recorded species and represented most of the floristic structure in the study area. However, eight other families accounted for 20% of the surveyed species, with two families (Apocynaceae and Solanaceae) represented by four species each (3.6%). Meanwhile, Brassicaceae and Cyperaceae were represented by three species (2.7%), and four families, Ebenaceae, Myrtaceae, Polygonaceae, and Rutaceae, were represented by two species each (1.8%). Eighteen families were monospecific, each with a single species (0.9%) (Figure S1, Table 1).

The most species-rich genera were *Euphorbia* L. (nine species, 8.18%) and *Ipomoea* L. (four species, 3.6%), while *Amaranthus* L. was represented by three species (2.7%). Nine genera were represented by two species each, and 76 genera were monotypic (1.08%). *Cynanchum acutum* L., *Cynodon dactylon*, *Echinochloa colona* (L.) Link and *Solanum nigrum* L. were the most common species across all communities (Table A1). Reclaimed desert lands were characterized by an abundance of stress-tolerant species, including *Amaranthus viridis* L., *Cyperus rotundus* L., *Megathyrsus maximus* (Jacq.) B. K. Simon & S. W. L. Jacobs, *Portulaca oleracea* L., *Caroxylon imbricatum* (Forssk.) Moq., *Setaria verticillata* (L.) P.Beauv., *Tamarix nilotica* (Ehrenb.) Bunge, and *Ziziphus spina-christi* (L.) Desf. In contrast, Nile Valley lands supported a distinct assemblage of more mesophytic and nitrophilous species, including *Bidens pilosa* L., *Urochloa reptans* (L.) Stapf, *Corchorus olitorius* L., *Erigeron bonariensis* L., *Euphorbia heterophylla* L., *Hibiscus tridactylites* Lindl., *Ipomoea cairica* (L.) Sweet, *Pluchea dioscoridis* (L.) DC., *Sida alba* L., *Sonchus oleraceus* L., and *Trianthema portulacastrum* L.

### 3.2. Life Form Structure of Sugarcane Weed Flora

The analysis of life form spectra reveals the adaptive strategies underlying the ecological differences in weed community composition across the two contrasting land types. The life forms of the collected weed species in the present study were classified into seven categories (Figure S2, Table A1). Therophytes were the most abundant life form, with 67 species, accounting for 60.91% of the total recorded species. Phanerophytes were represented by 20 species (18.18%), followed by Geophytes with eight species (7.27%), Hemicryptophytes were represented by seven species (6.36%), and Chamaephytes were represented by six species (5.45%). In contrast, the least-represented life-forms were Geophyte-Helophyte and Helophyte, which both attained a value of 0.91%.

### 3.3. Phytogeographical Affinities and Geographic Distribution of Weeds

The weed flora of Upper Egyptian sugarcane fields was dominated by broadly distributed chorotypes, with worldwide elements accounting for the largest proportion of recorded species (57 species; 51.82%). Neotropical elements constituted the largest regional group (23 species), followed by Palaeotropical (14 species), Cosmopolitan (13 species), and Pantropical elements (seven species). The pluriregional elements comprised 39 species (35.45%) with varying affinities, falling into 14 main chorotypes, of which 17 species are represented by the Irano-Turanian/Mediterranean/Saharo-Sindian/Sudano-Zambezian/Euro-Siberian chorotypes. Eight species represented the mono-regional chorotype (7.27%), with three species originating from the Sino-Japonic chorotype and two from Mediterranean chorotypes. The Australian, Irano-Turanian, and Sudano-Zambezian chorotypes were each represented by one species. Six species (5.45%) constituted the bi-regional chorotype, of which four were Saharo-Sindian/Sudano-Zambezian, and two were Sino-Japonic/Sudano-Zambezian (Figure S3, Table 2).

### 3.4. Spatial and Seasonal Patterns of Weed Diversity

**Figure 4:**
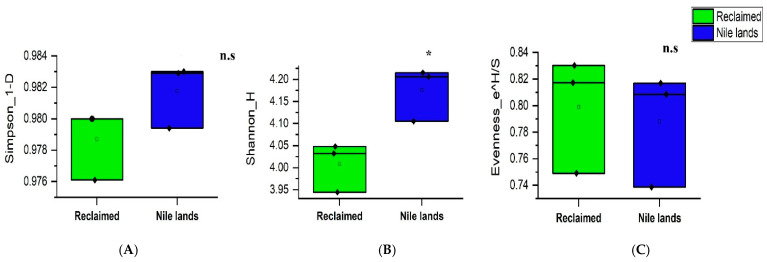
Comparison of plant community diversity indices between Reclaimed and Nile lands. The panels show (**A**) Simpson’s Diversity Index (1–D), (**B**) Shannon Diversity Index (H′), and (**C**) Evenness (e^H/S). Boxes represent the interquartile range (25th–75th percentiles), the horizontal line indicates the median, and symbols represent individual observations. An asterisk (*) denotes a statistically significant difference between land types (*p* < 0.05), whereas n.s. indicates a non-significant difference (*p* ≥ 0.05).

**Figure 5:**
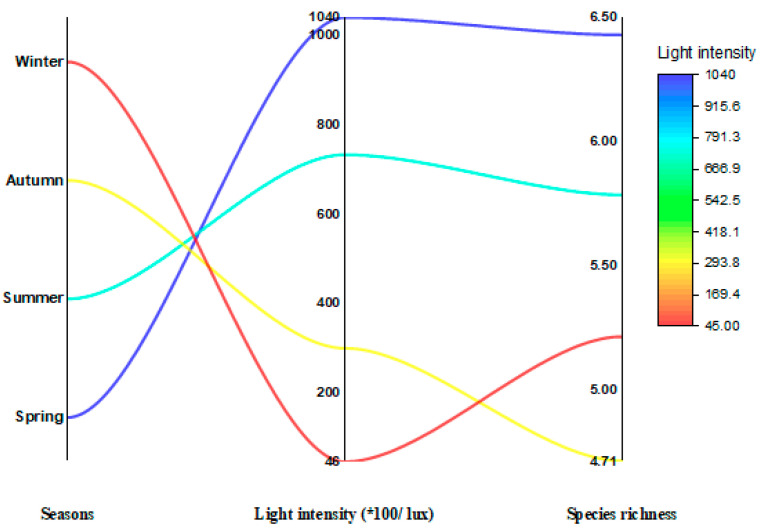
Seasonal variation in light intensity (×100 Lux) beneath the sugarcane canopy and mean weed species richness (species/stand) across four seasons in sugarcane fields of Qena Governorate, Upper Egypt (2024/2025).

Quantitative diversity indices confirmed significant differences in weed species richness and diversity between the Nile Valley and reclaimed desert lands. Simpson’s Index values were 0.97 for reclaimed land and 0.99 for Nile Valley land (*t* = 1.986, *p* = 0.118), indicating no significant difference in dominance structure between the two land types. However, Shannon-Wiener Index values differed significantly (H′ = 4.03 and 4.2, respectively; *t* = 3.945, *p* = 0.017), confirming that Nile Valley lands support significantly greater species richness and diversity. Evenness values were 0.83 and 0.81, respectively (*t* = 0.266, *p* = 0.804), indicating that neither land type is characterized by a single, overwhelmingly dominant species, despite their overall diversity differences (Figure 4). Seasonal variation in species richness was closely associated with changes in light intensity reaching the soil surface beneath the developing sugarcane canopy. Light intensity was highest in spring (1040 × 100 Lux) at the onset of sugarcane growth and declined progressively through summer and autumn as the canopy closed, reaching a minimum in winter (46 × 100 Lux). Species richness followed an identical trajectory, declining from 6.43 species/stand in spring to 5.79 in summer, reaching a minimum of 4.71 in autumn, before recovering slightly to 5.21 species/stand in winter (Figure 5).

### 3.5. Weed Community Composition and Structure

**Figure 6:**
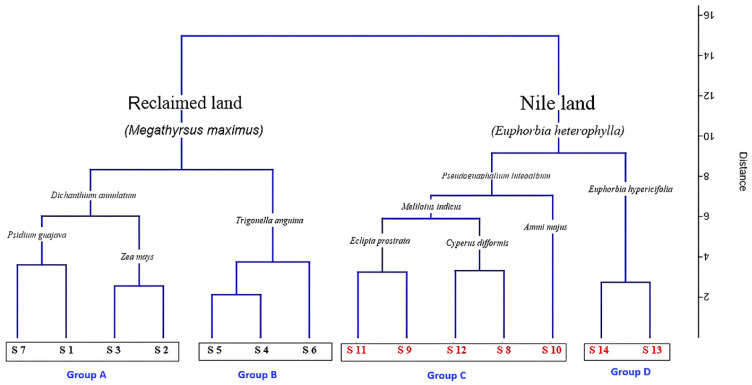
TWINSPAN hierarchical classification dendrogram of 14 sugarcane field stands (S1–S14) from reclaimed desert (S1–S7) and Nile Valley clay lands (S8–S14) in Qena Governorate, Upper Egypt. Stands separated into two main clusters, each subdivided into two sub-groups by indicator species at branching nodes.

The fourteen surveyed stands were separated into two main ecologically distinct weed communities (clusters), each associated with a characteristic soil environment and species assemblage, as revealed by TWINSPAN classification (Figure 6). Cluster 1 grouped the stands from reclaimed desert lands (S1–S7), while Cluster 2 grouped the stands from Nile lands (S8–S14). Within cluster 1, stands of the reclaimed desert lands were divided into two subgroups: subgroup A (stands 1, 2, 3, and 7) with *Dichanthium annulatum* as the indicator species, and subgroup B (stands 4, 5, and 6) with *Trigonella anguina* as the indicator species. Similarly, cluster 2 was divided into two subgroups: subgroup C (stands 8, 9, 10, 11, and 12) with *Pseudognaphalium luteoalbum* as the indicator species, and subgroup D (stands 13 and 14) with *Euphorbia hypericifolia* as the indicator species.

**Figure 7:**
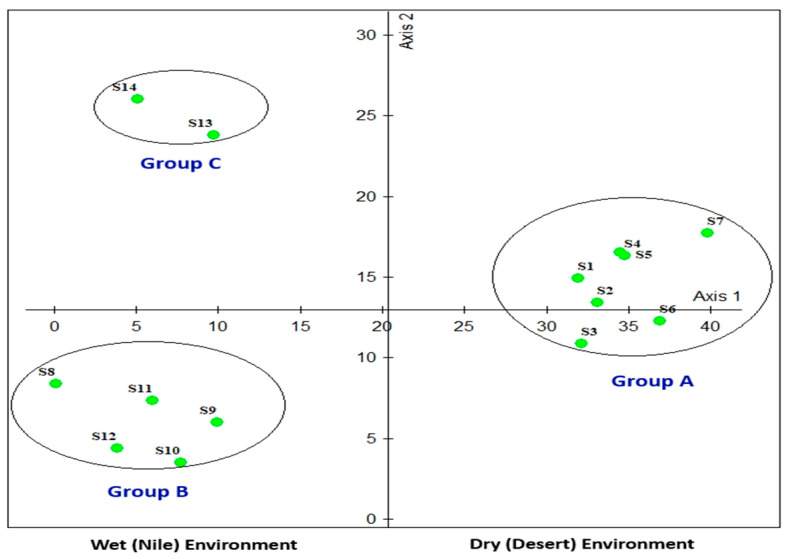
DCA ordination diagram of axes 1 and 2 for 14 sugarcane field stands (S1–S14) surveyed in Qena Governorate, Upper Egypt. A green dot represents an individual stand. The ordination reveals a distinct gradient along axis 1 related to elevation and proximity to the River Nile, along which the four TWINSPAN sub-groups aggregate into three ecologically coherent floristic groups (black open circles): Group A (reclaimed desert lands, S1–S7), Group B (Nile clay lands, S8–S12), and Group C (Nile riverbank lands, S13–S14).

The TWINSPAN clusters were arranged along DCA axes 1 and 2, revealing a distinct gradient related to elevation, nutrient availability, and proximity to the River Nile (Figure 7). The seven stands of the reclaimed land were aggregated into group A, forming a floristically coherent community defined by shared tolerance to saline and arid edaphic conditions characterized by stress-tolerant halophytic and xerophytic taxa including *Tamarix nilotica*, *Ziziphus spina-christi*, and *Salsola imbricata*, reflecting a community assembled primarily by physiological tolerance to high salinity (EC 2.60 mS/cm), elevated Na^+^ and CaCO_3_, and coarse sandy texture. On the other hand, group B comprised the five stands of the Nile clay land (S8–S12), representing a transitional community between the saline desert and fertile riverbank environments characterized by moderately fertile clay soils and by moisture-tolerant taxa, including *Eclipta prostrata* and *Cyperus difformis*. Group C comprised the two stands closest to the Nile riverbank (S13–S14), which supported the highest species richness (47.50 species/stand) and turnover (0.86 species/stand), with a community characterized by nitrophilous and moisture-demanding taxa, including *Euphorbia hypericifolia* and *Oxystelma esculentum*, reflecting the fertile, low-salinity, high organic matter conditions of sedimentary riverbank soils.

### 3.6. Edaphic Drivers of Weed Community Differentiation

**Figure 8:**
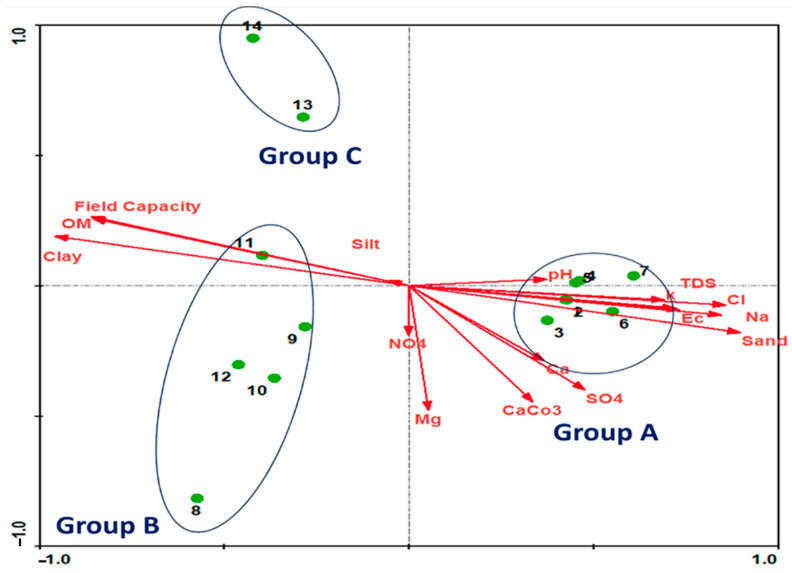
CCA biplot of 14 sugarcane field stands (S1–S14, green dots) and soil environmental variables (arrows) in Qena Governorate, Upper Egypt (2024/2025). Arrow length reflects variable importance; arrow direction indicates gradient orientation. Axis 1 separates Group A (reclaimed desert lands) from Groups B and C (Nile Valley lands), driven primarily by salinity variables (EC, Na, TDS, Cl) and soil texture (sand vs. clay and OM). Three floristic groups (A–C) are shown as black open circles. TDS, total dissolved solids; EC, electrical conductivity; OM, organic matter; Ca, calcium; Mg, magnesium; K, potassium; Na, sodium; Cl, chloride; CaCO_3_, calcium carbonate; SO_4_, sulfate; NO_3_, nitrate.

Canonical Correspondence Analysis confirmed that soil properties are the primary environmental variables structuring weed community composition across the studied stands. The CCA biplot confirmed the three floristic groups identified by DCA, positioning them along clear edaphic gradients (Figure 8). The eigenvalues of the first three CCA axes (0.390, 0.235, and 0.176) exceeded those of the corresponding unconstrained DCA axes (0.389, 0.178, and 0.054), indicating that the measured soil variables collectively account for a substantial proportion of the floristic variation among stands. The relatively high eigenvalue of CCA axis 1 (0.390) suggests that a single dominant edaphic gradient, primarily salinity and soil texture, explains most of the community differentiation, while axes 2 and 3 capture progressively smaller independent sources of floristic variation. DCA axis 1 correlated positively with Cl, K, Na, TDS, EC, and sand at *p* < 0.01 and negatively with organic matter, field capacity, and clay at *p* < 0.01 (Table S2).

Group A, comprising all seven reclaimed land stands, had the lowest species richness of the three communities (37.29 species/stand; turnover: 0.54 species/stand), consistent with the severe physiological constraints imposed by its highly saline, sandy-textured soils. This group is distinguished by the presence of *Amaranthus viridis*, *Cyperus rotundus*, *Megathyrsus maximus*, *Portulaca oleracea*, *Salsola imbricata*, *Setaria verticillata*, *Tamarix nilotica*, and *Ziziphus spina-christi* as indicator and dominant species. Compared with the other groups, Group A was associated with the highest levels of soil sodium, potassium, calcium, magnesium, sulfate, chloride, total dissolved salts, electrical conductivity, alkalinity (CaCO_3_), and sand (Table 3), but the lowest organic matter content, field capacity, clay, and silt, with soil texture ranging from loamy sand to sandy loam.

Group B comprised five Nile clay land stands (S8–S12) with intermediate species richness (41.6 species/stand; turnover: 0.54 species/stand), positioned ecologically between the saline desert community of Group A and the fertile riverbank community of Group C. *Eclipta prostrata*, *Cyperus difformis*, *Melilotus indicus*, and *Pseudognaphalium luteoalbum* characterized this group as indicator species. Soil samples exhibited lower ionic concentrations than those of Group A, but the highest silt content and a clay texture overall, representing the transitional community between Groups A and C across the edaphic gradient.

Group C comprised the two Nile riverbank stands (S13–S14), which supported the highest species richness (47.50 species/stand) and turnover (0.86 species/stand) of all three communities, reflecting the fertile, moist, low-salinity conditions of sedimentary riverbank soils. It is characterized by *Abelmoschus esculentus*, *Carica papaya*, *Euphorbia hypericifolia*, and *Oxystelma esculentum* as indicator species. This group had the lowest concentrations of most cations and anions, including sodium, chloride, and magnesium, and the highest organic matter, field capacity, and clay content, with clay as the dominant soil texture.

## 4. Discussion

### 4.1. Characteristics and Ecological Strategies of Sugarcane Weed Communities

Distribution, population density, and weed growth vary from one location to another based on soil, climate, and farmers’ management practices [42]. Understanding the relative contributions of edaphic heterogeneity and crop canopy dynamics to weed community assembly is essential for developing land-specific management strategies in irrigated agroecosystems. Our floristic survey across 14 sugarcane stands recorded 110 vascular plant species belonging to 33 families, of which Poaceae, Asteraceae, Fabaceae, Euphorbiaceae, and Amaranthaceae were the most species-rich, collectively representing about 54.55% of the total number of recorded weed species. These results agree with those of previous studies [3,43,44]. These families constitute the bulk of the flora in the study area across various habitats of the Nile River lands, including the north Nile Delta, the Nile Valley (Upper Egypt), and the northwestern part of the Nile Delta [45,46,47,48].

The dominance of annual species (61.82%) indicates low community stability and high compositional turnover, consistent with the highly disturbed nature of sugarcane agroecosystems. Annual species are characterized by high resource allocation to reproductive organs and early flowering, ensuring seed production even in shortened growing seasons [49]. Most perennial species, in contrast, lack the adaptations required for successful establishment in arable crops [50]. This community composition can change dramatically between seasons, depending on prevailing environmental conditions [51,52], a pattern particularly pronounced in the contrasting edaphic environments of reclaimed desert and Nile Valley lands.

*Cynanchum acutum*, *Cynodon dactylon*, *Echinochloa colonum*, and *Solanum nigrum* were the most common species in sugarcane communities, which agrees with the findings of Saeed et al. [53], who reported that *C. dactylon* and *C. acutum* were popular year-round weeds in reclaimed lands. *C. dactylon* is regarded as one of the worst weeds in the world, invades most of the temperate and subtropical agroecosystems, grows on practically all soil types, and can be a dangerous and serious invader that quickly spreads over cultivated land, making it difficult to eradicate [54,55]. In contrast, Leopardi-Verde et al. [56] found that the most abundant species in sugarcane cultivations in Colima, Mexico, were *Euphorbia hirta* and *Heliotropium procumbens*, while Lousada et al. [57] reported the dominance of *Cyperus rotundus* in sugarcane fields of Campos dos Goytacazes in the northern region of Rio de Janeiro State, Brazil, reflecting the influence of regional climate and soil conditions on weed assemblage identity.

The life form structure provides the necessary information to evaluate how vegetation responds to changes in environmental conditions. The life forms of the wild plant species recorded in the present study were grouped into seven types. Therophytes were the most abundant life form (60.91%), followed by Phanerophytes (18.18%), Geophytes (7.27%), Hemicryptophytes (6.36%), Chamaephytes (5.45%), Geophyte-Helophytes and Helophytes (0.91%). These results align with those of other studies [52,58]. In a study of weeds in different cereal crops in Egypt, Khalafallah et al. [59] found therophytes to be dominant. Galal [60] noted that therophytes are the predominant life forms, and most are weed species characteristic of cultivated lands. This finding coincides with Hassib’s [61] observation of Egyptian flora. Therophytes’ dominance can be explained by several factors, including their short life cycle and rapid growth rate, which allow them to withstand substrate instability, as well as their genetic and morphological adaptability to high levels of disturbances like light intensity, hot and dry climates, topographic variation, biotic factors, and human activity [62,63].

### 4.2. Biogeographic Origin and Floristic Affiliations

Egypt represents a biogeographical crossroads where floristic elements from at least four major regions intersect: the African Sudano-Zambezian, the Asiatic Irano-Turanian, the Afro-Asiatic Saharo-Arabian, and the Euro-Afro-Asiatic Mediterranean domains [2,64]. Phytogeographical affinities of the recorded species associated with sugarcane crops indicated that the dominance of the worldwide chorotype (51.82%) is represented by Neotropical, Palaeotropical, Cosmopolitan, and Pantropical elements (20.91%, 12.73%, 11.82%, and 6.36%, respectively). This indicates that human disturbances have a greater impact on the studied area’s floristic structure and are comparatively simpler than those of other parts of Egypt [65,66]. At the same time, bi- and pluriregional Mediterranean chorotypes were well represented, but pure Mediterranean species were underrepresented. The recorded flora was dominated by the Sudano-Zambezian chorotype, either pure or penetrated by other regions (mono-, bi-, and pluriregional). The diversity of Sudano-Zambezian plants reflects the tropical African origin of much of the desert flora and the flora’s ability to withstand extremely severe environmental conditions in these regions.

In contrast, Mediterranean plants are found in more temperate environments [67]. Comparable findings have been reported in other reclaimed regions in Upper Egypt [68,69] and in the Egyptian Oases by Abd El-Ghani and Fawzy [51]. On the other hand, these findings were consistent with previous studies on the flora of Upper Egypt, the Nile regions, and Nubia, which found that Sudano-Zambezian elements exceed Mediterranean elements across the entire flora [63,70].

### 4.3. Drivers of Spatial and Seasonal Variation in Weed Diversity

In our study, the three diversity indices (D, H′, and E) revealed a very high, well-balanced weed diversity in sugarcane fields in both reclaimed desert and Nile Valley lands: Simpson’s values near 1 and evenness values of 0.83 and 0.81 indicate low dominance and high evenness in both habitats [71]. On the other hand, Shannon–Wiener scores indicate higher diversity in sugarcane fields in the Nile Valley. In contrast, Simpson and evenness scores did not differ significantly, suggesting that cane fields of the Nile land host more species, whereas the overall dominance structure is similar across habitats [71,72]. This pattern (higher H’, similar D) is consistent with other studies, indicating that Simpson’s index is more sensitive to dominant species, whereas Shannon’s index gives more weight to species richness [71,72].

The slightly increased diversity and richness of sugarcane weeds in Nile lands may be due to the elevation gradient, nutrient-rich soils, and low salinity, which favor the establishment of a wide range of weed species adapted to moist, fertile environments. Our findings agree with Qian and Ricklefs [73], who noted that topography reduces similarity between regions or communities by increasing species’ spatial isolation and the heterogeneity of environmental factors, including microclimate. Moreover, the non-significant difference in Simpson’s Index, in contrast to the significant reduction in the Shannon–Wiener Index of sugarcane weeds on reclaimed land, indicates that land reclamation does not alter the dominance structure of weed communities but does reduce the richness of infrequent species [74,75]. This divergence implies that reclamation acts as an environmental filter, primarily removing the rare component of the flora; this pattern would have been missed if only Simpson’s Index had been used.

Seasonal variation in species richness was closely coupled with canopy light dynamics, declining progressively from a spring peak to an autumn minimum before partially recovering in winter. This pattern suggests that canopy closure progressively excludes light-demanding weed species as the crop develops, functioning as a temporally dynamic ecological filter that modulates weed diversity throughout the growing season [76,77]. The sugarcane canopy is least dense during early spring growth stages, favoring light-tolerant species and higher overall richness, whereas the increasingly shaded autumn understory suppresses many species. The partial recovery of richness in winter reflects the restoration of light availability following harvest or canopy senescence. This finding is consistent with Cheema et al. [78], who identified canopy competition as a crucial component of integrated weed management, reducing weed distribution during critical crop growth stages and thereby improving yield. Light availability, alongside other climatic variables, influences weed growth, reproduction, and dispersal in sugarcane plantations [79], with seasonal canopy development determining the intensity of this filtering effect throughout the growing cycle. Seasonal light attenuation therefore operates as a temporally dynamic ecological filter in combination with spatial edaphic differentiation, together producing the observed patterns of weed community assembly across Upper Egyptian sugarcane agroecosystems.

### 4.4. Phytosociological Community Assembly and Edaphic Drivers

Multivariate analyses have been widely applied to investigate relationships between soil properties and vegetation in arid and semi-arid habitats, consistently revealing pronounced edaphic gradients that structure floristic composition [45,80]. In the current study, three ecologically distinct weed communities were identified across the surveyed sugarcane stands, each associated with a characteristic edaphic environment. Group A consists of all stands of reclaimed lands representing desert environments with a high elevation, which had the lowest species richness and was distinguished by the presence of halophytic and xerophytic taxa, including *Amaranthus viridis*, *Cyperus rotundus*, *Megathyrsus maximus*, *Portulaca oleracea*, *Salsola imbricata Forssk*, *Setaria verticillata*, *Tamarix nilotica*, and *Ziziphus spina-christi*, whose co-occurrence reflects a community assembled primarily by physiological stress tolerance under the highest salinity, ionic concentration, and sand content recorded across all groups. This community is comparable to the stress-filtered assemblages reported by Salama et al. [45] in arid agroecological zones of the Nile Valley, and to similar vegetation groups documented in other crops across the Nile Valley [81].

On the other hand, group B involves five stands (S8–S12) of Nile lands, which are far away from the Nile bank border, representing a transitional community characterized by moisture-tolerant indicator species, including *Eclipta prostrata*, *Cyperus difformis*, *Melilotus indicus*, and *Pseudognaphalium luteoalbum*, reflecting intermediate edaphic conditions between the saline desert and the fertile riverbank environments. The moderately fertile clay soils of this group lower the physiological constraints imposed by salinity, permitting the establishment of a broader weed assemblage. Group C included the last two stands in Nile land along the Nile Riverbank, which had the lowest elevation, representing a wet, fertile environment and characterized by the highest species richness with the lowest concentration of most cations and anions, such as sodium, chloride, and magnesium, and the highest content of organic matter, field capacity, and clay. Its indicator species, *Abelmoschus esculentus*, *Carica papaya*, *Euphorbia hypericifolia*, and *Oxystelma esculentum* are characteristic of nitrophilous and moisture-demanding communities on fertile alluvial soils, reflecting community assembly driven by resource availability rather than stress tolerance.

These findings are consistent with those of Lousada et al. [57], who reported that weed density correlated with soil chemical and physical attributes, with clay content associated with greater weed density in sugarcane fields. Leon et al. [82] further demonstrated that soil texture significantly influences weed community structure by regulating nutrient availability, soil moisture, and light, with species richness highest in undisturbed field borders and lowest in rows and furrows. Similarly, Firehun and Tamado [9] pointed out that the main variables affecting weed dispersion in sugarcane communities were crop cycles, soil type, and fertilizer treatment.

The successive decreases in the eigenvalues of the first three CCA axes (0.390, 0.235, and 0.176) indicate that the measured soil variables meaningfully improve the explanation of floristic variation beyond species composition alone, confirming that they are unaffected environmental drivers of community differentiation rather than merely correlates. The relatively high eigenvalue of CCA axis 1 (0.390) indicates that a single dominant edaphic gradient, primarily salinity and soil texture, explains most of the community differentiation across the agroecological gradient, while axes 2 and 3 capture progressively smaller independent sources of floristic variation. This is consistent with vegetation ecology studies demonstrating that soil salinity is among the most powerful filters of plant community assembly in arid and semi-arid agricultural landscapes, where physiological tolerance to ionic stress determines species presence or absence more strongly than competitive interactions or dispersal limitation [83,84]. Comparable patterns have been reported across Egyptian agroecosystems, where bioclimatic region and crop type were the primary drivers of weed community variation, with older, fertile areas of the Nile Delta and the Nile Valley supporting richer weed floras than the recently reclaimed lands [81].

## 5. Conclusions

A two-tier environmental filtering mechanism regulates the assembly of weed communities in Upper Egyptian sugarcane fields. Soil type, defined primarily by electrical conductivity, sodium concentration, organic matter content, and clay fraction, operates as the dominant spatial filter, structuring three ecologically distinct communities along an edaphic gradient from saline reclaimed soils to fertile Nile riverbank alluvium. Overlaid upon this spatial framework, seasonal canopy-driven light attenuation acts as a temporally dynamic secondary filter, progressively excluding light-demanding species as the sugarcane canopy closes and partially restores richness during winter dormancy. Land reclamation reduces weed species richness by removing rare species from the flora without altering the community’s dominance structure, a distinction detectable only with complementary diversity indices and missed when Simpson’s Index is applied alone. The weed flora is dominated by cosmopolitan and Neotropical chorotypes, confirming that intensive agricultural management selects for broadly distributed, stress-tolerant, and ecologically plastic taxa over regionally endemic species. These findings establish that edaphic heterogeneity and canopy light dynamics are not independent drivers but interact hierarchically to determine weed community composition in irrigated subtropical agroecosystems. Effective weed management in contrasting land types, therefore, requires land-specific strategies: salinity-targeted interventions in reclaimed desert lands and canopy management to exploit light competition in Nile Valley fields.

## Figures and Tables

**Table 1:** Number of weed species and their corresponding plant families recorded in sugarcane fields in Qena Governorate, Upper Egypt.

Family Name	Number of Species	Percentage of Total Flora (%)
Aizoaceae	1	0.9
Amaranthaceae	8	7.3
Amaryllidaceae	1	0.9
Anacardiaceae	1	0.9
Apiaceae	1	0.9
Apocynaceae	4	3.6
Arecaceae	1	0.9
Asteraceae	12	10.9
Brassicaceae	3	2.7
Caricaceae	1	0.9
Convolvulaceae	5	4.5
Combretaceae	1	0.9
Cucurbitaceae	3	2.7
Cyperaceae	2	1.8
Ebenaceae	1	0.9
Euphorbiaceae	10	9.1
Fabaceae	11	10.0
Malvaceae	5	4.5
Myrtaceae	2	1.8
Oleaceae	1	0.9
Oxalidaceae	1	0.9
Pedaliaceae	1	0.9
Plantaginaceae	1	0.9
Poaceae	19	17.3
Polygonaceae	2	1.8
Portulacaceae	1	0.9
Rhamnaceae	1	0.9
Rutaceae	2	1.8
Salicaceae	1	0.9
Solanaceae	4	3.6
Tamaricaceae	1	0.9
Typhaceae	1	0.9
Zygophyllaceae	1	0.9
**Total**	**110**	**100**

**Table 2:** Number of species and percentage of various floristic categories in the study area. Chorotype abbreviations: CAP: Capensis; OSM: Cosmopolitan; ES: Euro-Siberian; GC: Guino-Congo; ME: Mediterranean; PAL: Palaeotropical; PAN: Pantropical; IT: Irano-Turanian; SS: Saharo-Sindian; SJ: Sino-Japonic; SZ: Sudano-Zambezian.

No.	Floristic Category	Total Area		Geographical Distribution
		No.	%	
1	PAN	7	6.36	Worldwide
2	PAL	14	12.73	
3	NEO	23	20.91	
4	COSM	13	11.82	
5	SJ	3	2.73	Mono-regional elements
6	ME	2	1.82	
7	AUS	1	0.91	
8	IT	1	0.91	
9	SZ	1	0.91	
10	SS + SZ	4	3.64	Bi-regional elements
11	SJ + SZ	2	1.82	
12	ME + SS + SZ	1	0.91	Pluri-regional elements
13	IT + ME + SJ	1	0.91	
14	IT + SS + SZ	2	1.82	
15	IT + ME + SS	2	1.82	
16	SJ + SS + SZ	1	0.91	
17	IT + ME + SS + SZ	12	10.91	
18	IT + ME + SJ + SS	2	1.82	
19	ES + IT + ME + SS	1	0.91	
20	IT + ME + SA + SZ	2	1.82	
21	ES + IT + ME + SS + SZ	7	6.36	
22	ES + IT + ME + SJ + SS	2	1.82	
23	ES + IT + ME + SJ + SS + SZ	1	0.91	
24	CAP + GC + ME + IT + SS + SZ	3	2.73	
25	IT + ME + SJ + SS + SZ	2	1.82	
	**Total**	**110**	**100**	

**Table 3:** Mean values (±standard deviations, SDs) and ANOVA results of the soil variables across DCA vegetation Groups (A–C) in the study area. Different superscript lowercase letters in the same row indicate significant differences in soil parameters among groups (*p* ≤ 0.05).

	Group A	Group B	Group C
Cl^−^ (mg/g)	3.99 ± 1.83 ^a^	0.46 ± 0.05 ^b^	0.38 ± 0.04 ^b^
SO_4_^2−^ (mg/g)	1.01 ± 0.25 ^a^	0.88 ± 0.14 ^a^	0.57 ± 0.12 ^b^
NO_3_^−^ (mg/g)	0.05 ± 0.05 ^a^	0.07 ± 0.08 ^a^	0.01 ± 0.00 ^a^
CaCO_3_ (mg/g)	8.86 ± 0.36 ^a^	8.37 ± 2.44 ^a^	6.44 ± 0.06 ^b^
K^+^ (mg/g)	0.45 ± 0.38 ^a^	0.05 ± 0.03 ^b^	0.01 ± 0.00 ^b^
Na^+^ (mg/g)	1.21 ± 0.58 ^a^	0.11 ^b^	0.18 ± 0.01 ^b^
Ca^2+^ (mg/g)	1.33 ± 1.25 ^a^	0.68 ± 0.53 ^a^	0.40 ± 0.04 ^a^
Mg^2+^ (mg/g)	0.23 ± 0.15 ^a^	0.20 ± 0.20 ^a^	0.13 ± 0.01 ^a^
pH	7.89 ± 0.10 ^a^	7.80 ± 0.31 ^ab^	7.64 ± 0.09 ^b^
TDS (g/L)	1.69 ± 1.20 ^a^	0.30 ± 0.09 ^b^	0.21 ± 0.00 ^b^
EC (mS/cm)	2.60 ± 1.79 ^a^	0.50 ± 0.14 ^b^	0.33 ± 0.01 ^b^
Organic matter %	4.56 ± 2.60 ^a^	12.98 ± 1.07 ^b^	13.80 ± 0.57 ^b^
Field Capacity %	9.49 ± 6.40 ^a^	29.17 ± 2.19 ^b^	31.90 ± 2.11 ^b^
Clay %	5.13 ± 3.68 ^a^	54.46 ± 3.56 ^b^	69.10 ± 0.57 ^c^
Silt %	15.10 ± 3.02 ^a^	18.76 ± 12.58 ^a^	18.10 ± 5.80 ^a^
Sand %	79.77 ± 5.30 ^a^	26.78 ± 10.80 ^b^	12.80 ± 5.23 ^c^

## Data Availability

The raw data supporting the conclusions of this article will be made available by the authors on request.

## Supplementary Materials

The following supporting information can be downloaded at: https://www.mdpi.com/article/10.3390/plants15121825/s1, Figure S1. Percentage of the plant families recorded in sugar cane fields in Qena Governorate, Upper Egypt; Figure S2. A chord diagram visualizing the association between Raunkiaer’s life forms and the plant families recorded in the study area. The width of the connecting chords represents the proportional number of species shared between each life form and related plant family; Figure S3. A chord diagram displaying the relationship between chorotypes and plant families recorded in sugarcane fields in Qena Governorate, Egypt; Table S1: ID number for the voucher specimens deposited at the Herbarium of Faculty of Science, Qena University, Egypt. Species are arranged alphabetically; Table S2: Simple linear correlation coefficient (r) between the estimated soil variables and DCA axes 1 and 2, * *p* < 0.05, ** *p* < 0.01.

## Abbreviations

The following abbreviations are used in this manuscript:

CCA	Canonical Correspondence Analysis
DCA	Detrended Correspondence Analysis
TWINSPAN	Two-way indicator species analysis

## Appendix A

**Table A1:** List of plant species recorded in sugarcane fields, along with their families, growth forms, life spans, and chorotypes. Chorotype abbreviations: CAP: Capensis; COSM: Cosmopolitan; ES: Euro-Siberian; GC: Guino-Congo; ME: Mediterranean; PAL: Palaeotropical; PAN: Pantropical; IT: Irano-Turanian; SS: Saharo-Sindian; SJ: Sino-Japonic; SZ: Sudano-Zambezian.

Family	Taxa	Life Span	Life Form	Chorotype	Frequency
**Aizoaceae**	*Trianthema portulacastrum* L.	Annual	Therophyte	PAN	92.86
**Amaranthaceae**	*Amaranthus graecizans* L. subsp. *graecizans*	Annual	Therophyte	PAL	7.14
	*Amaranthus retroflexus* L.	Annual	Therophyte	NEO	35.71
	*Amaranthus viridis* L.	Annual	Therophyte	NEO	92.86
	*Caroxylon imbricatum* (Forssk.) Moq.	Perennial	Chamaephyte	IT + ME + SS + SZ	50.00
	*Bassia muricata* (L.) Asch.	Annual	Therophyte	IT + ME + SS + SZ	42.86
	*Beta vulgaris* subsp. *vulgaris*	Annual	Therophyte	ES + IT + ME + SS + SZ	7.14
	*Chenopodium album* L.	Annual	Therophyte	COSM	64.29
	*Chenopodiastrum murale* (L.) S.Fuentes, Uotila & Borsch	Annual	Therophyte	ES + IT + ME + SS + SZ	64.29
**Amaryllidaceae**	*Allium cepa* L.	Perennial	Geophyte	IT	7.14
**Anacardiaceae**	*Mangifera indica* L.	Perennial	Phanerophyte	SJ	7.14
**Apiaceae**	*Ammi majus* L.	Annual	Therophyte	ES + IT + ME + SS + SZ	14.29
**Apocynaceae**	*Calotropis procera* (Aiton) W.T.Aiton	Perennial	Phanerophyte	IT + ME + SJ + SS	35.71
	*Oxystelma esculentum* (L.f.) Sm.	Perennial	Hemicryptophyte	PAN	7.14
	*Cynanchum acutum* L.	Perennial	Chamaephyte	ES + IT + ME + SJ + SS	100
	*Leptadenia arborea* (Forssk.) Schweinf.	Perennial	Phanerophyte	SS + SZ	7.14
**Arecaceae**	*Phoenix dactylifera* L.	Perennial	Phanerophyte	IT + SS + SZ	78.57
**Asteraceae**	*Bidens pilosa* L.	Annual	Therophyte	NEO	64.29
	*Eclipta prostrata* (L.) L.	Annual	Therophyte	NEO	21.43
	*Erigeron bonariensis* L.	Annual	Therophyte	NEO	78.57
	*Lactuca serriola* L.	Annual	Therophyte	ES + IT + ME + SS + SZ	42.86
	*Launaea mucronata* subsp. *cassiniana* (Jaub. & Spach) N.Kilian	Perennial	Hemicryptophyte	IT + ME + SS + SZ	14.29
	*Launaea nudicaulis* (L.) Hook.f.	Perennial	Hemicryptophyte	IT + ME + SS + SZ	21.43
	*Pluchea dioscoridis* (L.) DC.	Perennial	Phanerophyte	SS + SZ	64.29
	*Sonchus oleraceus* L.	Annual	Therophyte	ES + IT + ME + SS + SZ	92.86
	*Symphyotrichum squamatum* (Spreng.) G.L.Nesom	Perennial	Chamaephyte	NEO	21.43
	*Urospermum picroides* (L.) Scop. ex F.W.Schmidt	Annual	Therophyte	IT + ME + SS + SZ	7.14
	*Pseudognaphalium luteoalbum* (L.) Hilliard & B.L.Burtt	Annual	Therophyte	COSM	35.71
	*Xanthium strumarium* L.	Annual	Therophyte	PAL	28.57
**Brassicaceae**	*Brassica rapa* L.	Annual	Therophyte	IT + ME + SS + SZ	42.86
	*Eruca sativa* Mill.	Annual	Therophyte	IT + ME + SJ + SS	28.57
	*Raphanus raphanistrum* subsp. *sativus* (L.) Schmalh.	Annual	Therophyte	ME	7.14
**Caricaceae**	*Carica papaya* L.	Perennial	Phanerophyte	NEO	7.14
**Convolvulaceae**	*Convolvulus arvensis* L.	Perennial	Hemicryptophyte	PAL	71.43
	*Ipomoea carnea* Jacq.	Perennial	Phanerophyte	NEO	14.29
	*Ipomoea eriocarpa* R.Br.	Annual	Therophyte	PAN	14.29
	*Ipomoea cairica* (L.) Sweet	Perennial	Geophyte	PAL	92.86
	*Ipomoea triloba* L.	Annual	Therophyte	NEO	71.43
**Combretaceae**	*Conocarpus erectus* L.	Perennial	Phanerophyte	PAN	21.43
**Cucurbitaceae**	*Citrullus lanatus* (Thunb.) Matsum. & Nakai	Annual	Therophyte	ME + SS + SZ	14.29
	*Cucumis melo* L.	Annual	Therophyte	PAL	35.71
	*Cucumis sativus* L.	Annual	Therophyte	SJ	28.57
**Cyperaceae**	*Cyperus difformis* L.	Annual	Therophyte	COSM	14.29
	*Cyperus rotundus* L.	Perennial	Geophyte	COSM	92.86
**Ebenaceae**	*Diospyros kaki* Thunb.	Perennial	Phanerophyte	SJ	7.14
**Euphorbiaceae**	*Euphorbia heterophylla* L.	Annual	Therophyte	NEO	50.00
	*Euphorbia hirta* L.	Annual	Therophyte	NEO	35.71
	*Euphorbia hypericifolia* L.	Annual	Therophyte	NEO	14.29
	*Euphorbia indica* Lam.	Annual	Therophyte	IT + ME + SJ	7.14
	*Euphorbia maculata* L.	Annual	Therophyte	NEO	14.29
	*Euphorbia nutans* Lag.	Annual	Therophyte	NEO	14.29
	*Euphorbia peplus* L.	Annual	Therophyte	ES + IT + ME + SS + SZ	21.43
	*Euphorbia prostrata* Aiton	Annual	Therophyte	NEO	7.14
	*Euphorbia serpens* Kunth	Annual	Therophyte	NEO	14.29
	*Ricinus communis* L.	Perennial	Phanerophyte	SZ	28.57
**Fabaceae**	*Vachellia nilotica* (L.) P.J.H.Hurter & Mabb.	Perennial	Phanerophyte	IT + SS + SZ	28.57
	*Alhagi graecorum* Boiss.	Perennial	Chamaephyte	IT + ME + SS + SZ	28.57
	*Lotus arabicus* Sol. ex L.	Annual	Therophyte	SS + SZ	7.14
	*Medicago sativa* L.	Perennial	Hemicryptophyte	ES + IT + ME + SS	21.43
	*Melilotus indicus* (L.) All.	Annual	Therophyte	COSM	78.57
	*Leucaena leucocephala* (Lam.) de Wit	Perennial	Phanerophyte	NEO	7.14
	*Trigonella anguina* Delile	Annual	Therophyte	IT + ME + SS + SZ	35.71
	*Trifolium alexandrinum* L.	Annual	Therophyte	IT + ME + SS	35.71
	*Trifolium resupinatum* L.	Annual	Therophyte	IT + ME + SS	7.14
	*Sesbania sesban* (L.) Merr.	Perennial	Phanerophyte	PAN	71.43
	*Vicia faba* L.	Annual	Therophyte	ME	7.14
**Malvaceae**	*Abelmoschus esculentus* (L.) Moench	Annual	Therophyte	SJ + SZ	7.14
	*Corchorus olitorius* L.	Annual	Therophyte	PAL	92.86
	*Hibiscus tridactylites* Lindl.	Annual	Therophyte	PAL + AUS	57.14
	*Malva parviflora* L.	Annual	Therophyte	IT + ME + SS + SZ	64.29
	*Sida alba* L.	Annual	Hemicryptophyte	SS + SZ	85.71
**Myrtaceae**	*Eucalyptus camaldulensis* Dehnh.	Perennial	Phanerophyte	AUS	42.86
	*Psidium guajava* L.	Perennial	Phanerophyte	NEO	14.29
**Oleaceae**	*Olea europaea* L.	Perennial	Phanerophyte	CAP + GC + ME + IT + SS + SZ	28.57
**Oxalidaceae**	*Oxalis corniculata* L.	Annual	Therophyte	SJ + SS + SZ	21.43
**Pedaliaceae**	*Sesamum indicum* L.	Annual	Therophyte	SJ + SZ	14.29
**Plantaginaceae**	*Plantago major* L.	Perennial	Hemicryptophyte	COSM	28.57
**Poaceae**	*Avena fatua* L.	Annual	Therophyte	COSM	21.43
	*Cenchrus echinatus* L.	Annual	Therophyte	NEO	7.14
	*Cynodon dactylon* (L.) Pers.	Perennial	Geophyte	COSM	100
	*Dactyloctenium aegyptium* (L.) Willd.	Annual	Therophyte	PAL	50.00
	*Desmostachya bipinnata* (L.) Stapf	Perennial	Geophyte	PAL	28.57
	*Dichanthium annulatum* (Forssk.) Stapf	Perennial	Geophyte	PAL	42.86
	*Digitaria sanguinalis* (L.) Scop.	Annual	Therophyte	COSM	7.14
	*Echinochloa colona* (L.) Link	Annual	Therophyte	PAL	100
	*Imperata cylindrica* (L.) Raeusch.	Perennial	Geophyte	CAP + GC + ME + IT + SS + SZ	42.86
	*Megathyrsus maximus* (Jacq.) B.K.Simon & S.W.L.Jacobs	Perennial	Geophyte	PAL	50.00
	*Phragmites australis* (Cav.) Trin. ex Steud.	Perennial	Geophyte-Helophyte	COSM	85.71
	*Polypogon monspeliensis* (L.) Desf.	Annual	Therophyte	IT + ME + SJ + SS + SZ	7.14
	*Setaria verticillata* (L.) P.Beauv.	Annual	Therophyte	COSM	78.57
	*Triticum aestivum* L.	Annual	Therophyte	IT + ME + SS + SZ	35.71
	*Urochloa ramosa* (L.) T.Q.Nguyen	Annual	Therophyte	PAL	14.29
	*Urochloa reptans* (L.) Stapf	Annual	Therophyte	PAN	64.29
	*Sorghum bicolor* (L.) Moench	Annual	Therophyte	PAL	7.14
	*Sorghum virgatum* (Hack.) Stapf	Annual	Therophyte	IT + ME + SA + SZ	7.14
	*Zea mays* L.	Annual	Therophyte	NEO	28.57
**Polygonaceae**	*Rumex dentatus* L.	Annual	Therophyte	ES + IT + ME + SJ + SS	28.57
	*Rumex spinosus* L.	Annual	Therophyte	IT + ME + SA + SZ	28.57
**Portulacaceae**	*Portulaca oleracea* L.	Annual	Therophyte	ES + IT + ME + SS + SZ	92.86
**Rhamnaceae**	*Ziziphus spina-christi* (L.) Desf.	Perennial	Phanerophyte	IT + ME + SS + SZ	85.71
**Rutaceae**	*Casimiroa edulis* La Llave	Perennial	Phanerophyte	NEO	7.14
	*Citrus* × *limon* (L.) Osbeck	Perennial	Phanerophyte	PAN	7.14
**Salicaceae**	*Salix mucronata* Thunb.	Perennial	Phanerophyte	CAP + GC + ME + IT + SS + SZ	14.29
**Solanaceae**	*Solanum lycopersicum* L.	Annual	Therophyte	NEO	14.29
	*Solanum nigrum* L.	Annual	Therophyte	COSM	100
	*Physalis angulata* L.	Annual	Therophyte	NEO	28.57
	*Withania somnifera* (L.) Dunal	Perennial	Chamaephyte	IT + ME + SJ + SS + SZ	7.14
**Tamaricaceae**	*Tamarix nilotica* (Ehrenb.) Bunge	Perennial	Phanerophyte	IT + ME + SS + SZ	50.00
**Typhaceae**	*Typha domingensis* Pers.	Perennial	Helophyte	COSM	7.14
**Zygophyllaceae**	*Tribulus terrestris* L.	Annual	Therophyte	ES + IT + ME + SJ + SS + SZ	14.29
