# Supplementary material for: Environmental Drivers of Weed Floristic Diversity in Two Contrasting Sugarcane Agroecosystems
Source: Plants (Basel). 2026 Jun 12;15(12):1825. doi: 10.3390/plants15121825 (PMC13306420; doi:10.3390/plants15121825)
Supplement: Supplementary file 1 [file plants-15-01825-s001.zip › plants-4307430-supplementary.pdf]

SUPPLEMENTARY MATERIALS:

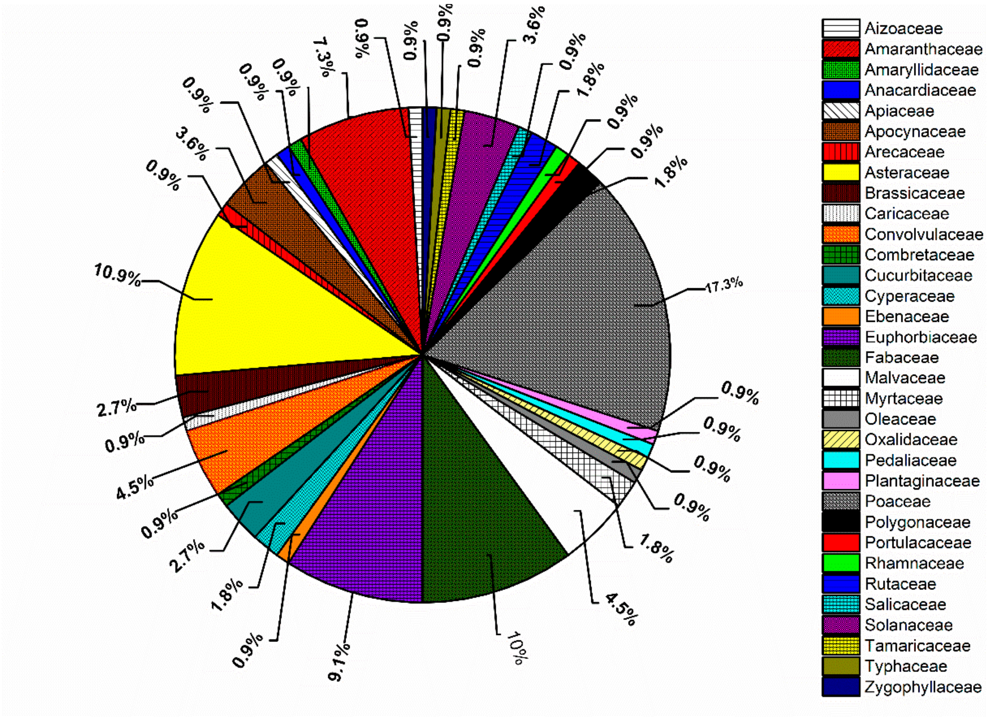

Figure S1. Percentage of the plant families recorded in sugar cane fields at Qena Governorate, Upper Egypt.

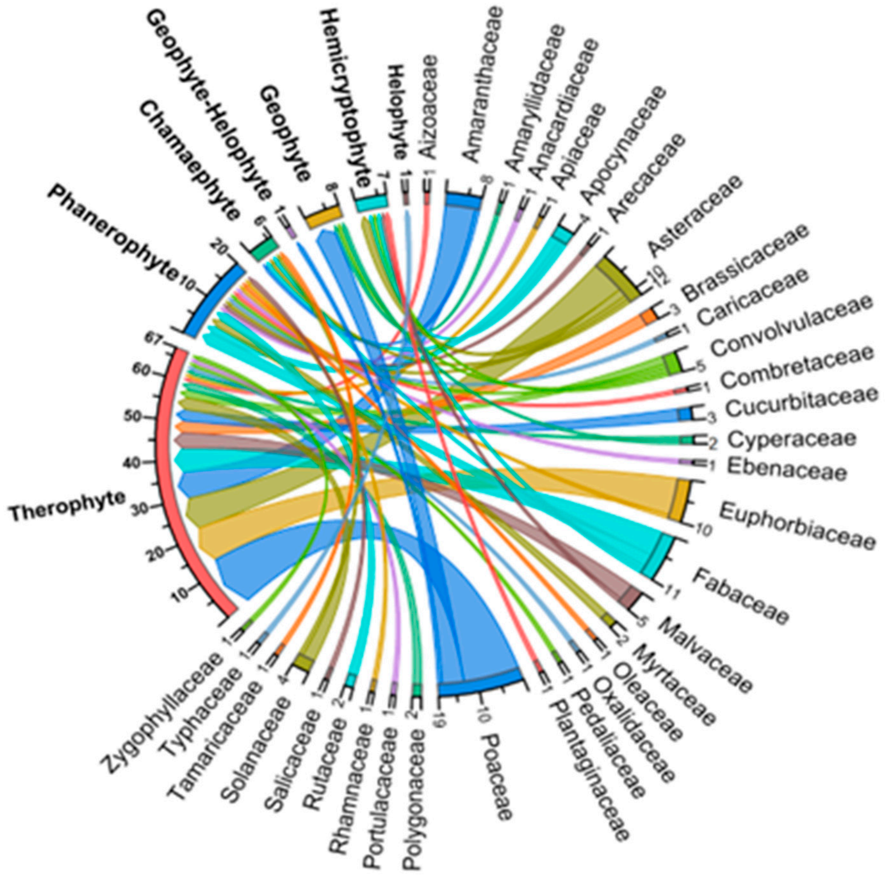

Figure S2. A chord diagram visualizes the association between Raunkiaer's life forms and the plant families recorded in the study area. The width of the connecting chords represents the proportional number of species shared between each life form and related plant family.

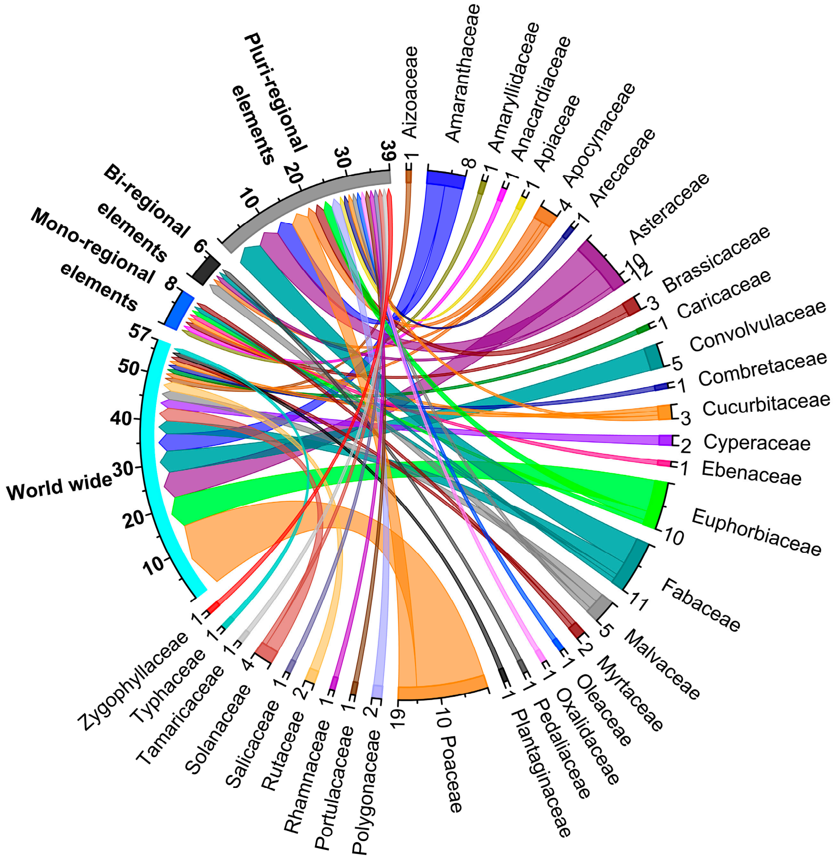

Figure S3. A chord diagram displaying the relationship between chorotypes and plant families recorded in sugarcane fields at Qena Governorate, Egypt.

Table S1. ID number for the voucher specimens deposited at the Herbarium of Faculty of Science, Qena University, Egypt. Species are arranged alphabetically.

Specimen name	Accession Number	Collector	Date of collection
Abelmoschus esculentus (L.) Moench	QNA-2091	Osman, A. K & Mousa, M.A.	13/8/2024
Alhagi graecorum Boiss.	QNA-2001	Osman, A. K & Mousa, M.A.	23/5/2024
Allium cepa L.	QNA-2002	Osman, A. K & Mousa, M.A.	23/5/2024
Amaranthus graecizans L. subsp. *graecizans*	QNA-2003	Osman, A. K & Mousa, M.A.	23/5/2024
Amaranthus retroflexus L.	QNA-2092	Osman, A. K & Mousa, M.A.	13/8/2024
Amaranthus viridis L.	QNA-2004	Osman, A. K & Mousa, M.A.	23/5/2024
Ammi majus L.	QNA-2005	Osman, A. K & Mousa, M.A.	23/5/2024
Avena fatua L.	QNA-2102	Osman, A. K & Mousa, M.A.	15/2/2025
Bassia muricata (L.) Asch.	QNA-2006	Osman, A. K & Mousa, M.A.	23/5/2024
Beta vulgaris subsp. *vulgaris*	QNA-2103	Osman, A. K & Mousa, M.A.	15/2/2025
Bidens pilosa L.	QNA-2007	Osman, A. K & Mousa, M.A.	23/5/2024
Brassica rapa L.	QNA-2093	Osman, A. K & Mousa, M.A.	13/8/2024
Calotropis procera (Aiton) W.T.Aiton	QNA-2008	Osman, A. K & Mousa, M.A.	23/5/2024
Carica papaya L.	QNA-2009	Osman, A. K & Mousa, M.A.	23/5/2024
Caroxylon imbricatum (Forssk.) Moq.	QNA-2010	Osman, A. K & Mousa, M.A.	23/5/2024
Casimiroa edulis La Llave	QNA-2011	Osman, A. K & Mousa, M.A.	23/5/2024
Cenchrus echinatus L.	QNA-2012	Osman, A. K & Mousa, M.A.	23/5/2024
Chenopodiastrum murale (L.) S.Fuentes, Uotila & Borsch	QNA-2013	Osman, A. K & Mousa, M.A.	23/5/2024
Chenopodium album L.	QNA-2014	Osman, A. K & Mousa, M.A.	23/5/2024
Citrullus lanatus (Thunb.) Matsum. & Nakai	QNA-2015	Osman, A. K & Mousa, M.A.	23/5/2024
Citrus × *limon* (L.) Osbeck	QNA-2016	Osman, A. K & Mousa, M.A.	23/5/2024

Conocarpus erectus L.	QNA-2017	Osman, A. K & Mousa, M.A.	23/5/2024
Convolvulus arvensis L.	QNA-2018	Osman, A. K & Mousa, M.A.	23/5/2024
Corchorus olitorius L.	QNA-2019	Osman, A. K & Mousa, M.A.	23/5/2024
Cucumis melo L.	QNA-2020	Osman, A. K & Mousa, M.A.	23/5/2024
Cucumis sativus L.	QNA-2021	Osman, A. K & Mousa, M.A.	23/5/2024
Cynanchum acutum L.	QNA-2022	Osman, A. K & Mousa, M.A.	23/5/2024
Cynodon dactylon (L.) Pers.	QNA-2023	Osman, A. K & Mousa, M.A.	23/5/2024
Cyperus difformis L.	QNA-2104	Osman, A. K & Mousa, M.A.	15/2/2025
Cyperus rotundus L.	QNA-2024	Osman, A. K & Mousa, M.A.	23/5/2024
Dactyloctenium aegyptium (L.) Willd.	QNA-2025	Osman, A. K & Mousa, M.A.	23/5/2024
Desmostachya bipinnata (L.) Stapf	QNA-2026	Osman, A. K & Mousa, M.A.	23/5/2024
Dichanthium annulatum (Forssk.) Stapf	QNA-2027	Osman, A. K & Mousa, M.A.	23/5/2024
Digitaria sanguinalis (L.) Scop.	QNA-2028	Osman, A. K & Mousa, M.A.	23/5/2024
Diospyros kaki Thunb.	QNA-2029	Osman, A. K & Mousa, M.A.	23/5/2024
Echinochloa colona (L.) Link	QNA-2030	Osman, A. K & Mousa, M.A.	23/5/2024
Eclipta prostrata (L.) L.	QNA-2031	Osman, A. K & Mousa, M.A.	23/5/2024
Erigeron bonariensis L.	QNA-2032	Osman, A. K & Mousa, M.A.	23/5/2024
Eruca sativa Mill.	QNA-2033	Osman, A. K & Mousa, M.A.	23/5/2024
Eucalyptus camaldulensis Dehnh.	QNA-2034	Osman, A. K & Mousa, M.A.	23/5/2024
Euphorbia heterophylla L.	QNA-2035	Osman, A. K & Mousa, M.A.	23/5/2024
Euphorbia hirta L.	QNA-2036	Osman, A. K & Mousa, M.A.	23/5/2024
Euphorbia hypericifolia L.	QNA-2037	Osman, A. K & Mousa, M.A.	23/5/2024
Euphorbia indica Lam.	QNA-2038	Osman, A. K & Mousa, M.A.	23/5/2024
Euphorbia maculata L.	QNA-2094	Osman, A. K & Mousa, M.A.	13/8/2024

Euphorbia nutans Lag.	QNA-2095	Osman, A. K & Mousa, M.A.	13/8/2024
Euphorbia peplus L.	QNA-2096	Osman, A. K & Mousa, M.A.	13/8/2024
Euphorbia prostrata Aiton	QNA-2105	Osman, A. K & Mousa, M.A.	15/2/2025
Euphorbia serpens Kunth	QNA-2039	Osman, A. K & Mousa, M.A.	23/5/2024
Hibiscus tridactylites Lindl.	QNA-2040	Osman, A. K & Mousa, M.A.	23/5/2024
Imperata cylindrica (L.) Raeusch.	QNA-2041	Osman, A. K & Mousa, M.A.	23/5/2024
Ipomoea cairica (L.) Sweet	QNA-2042	Osman, A. K & Mousa, M.A.	23/5/2024
Ipomoea carnea Jacq.	QNA-2043	Osman, A. K & Mousa, M.A.	23/5/2024
Ipomoea eriocarpa R.Br.	QNA-2106	Osman, A. K & Mousa, M.A.	15/2/2025
Ipomoea triloba L.	QNA-2044	Osman, A. K & Mousa, M.A.	23/5/2024
Lactuca serriola L.	QNA-2045	Osman, A. K & Mousa, M.A.	23/5/2024
Launaea mucronata subsp. *cassiniana* (Jaub . & Spach) N.Kilian	QNA-2107	Osman, A. K & Mousa, M.A.	15/2/2025
Launaea nudicaulis (L.) Hook.f.	QNA-2046	Osman, A. K & Mousa, M.A.	23/5/2024
Leptadenia arborea (Forssk.) Schweinf.	QNA-2108	Osman, A. K & Mousa, M.A.	15/2/2025
Leucaena leucocephala (Lam.) de Wit	QNA-2047	Osman, A. K & Mousa, M.A.	23/5/2024
Lotus arabicus Sol. ex L.	QNA-2048	Osman, A. K & Mousa, M.A.	23/5/2024
Malva parviflora L.	QNA-2049	Osman, A. K & Mousa, M.A.	23/5/2024
Mangifera indica L.	QNA-2050	Osman, A. K & Mousa, M.A.	23/5/2024
Medicago sativa L.	QNA-2051	Osman, A. K & Mousa, M.A.	23/5/2024
Megathyrsus maximus (Jacq.) B.K.Simon & S.W.L.Jacobs	QNA-2052	Osman, A. K & Mousa, M.A.	23/5/2024
Melilotus indicus (L.) All.	QNA-2109	Osman, A. K & Mousa, M.A.	15/2/2025
Olea europaea L.	QNA-2053	Osman, A. K & Mousa, M.A.	23/5/2024
Oxalis corniculata L.	QNA-2054	Osman, A. K & Mousa, M.A.	23/5/2024

Oxystelma esculentum (L.f.) Sm.	QNA-2055	Osman, A. K & Mousa, M.A.	23/5/2024
Phoenix dactylifera L.	QNA-2056	Osman, A. K & Mousa, M.A.	23/5/2024
Phragmites australis (Cav.) Trin. ex Steud.	QNA-2057	Osman, A. K & Mousa, M.A.	23/5/2024
Physalis angulata L.	QNA-2058	Osman, A. K & Mousa, M.A.	23/5/2024
Plantago major L.	QNA-2097	Osman, A. K & Mousa, M.A.	13/8/2024
Pluchea dioscoridis (L.) DC.	QNA-2059	Osman, A. K & Mousa, M.A.	23/5/2024
Polypogon monspeliensis (L.) Desf.	QNA-2060	Osman, A. K & Mousa, M.A.	23/5/2024
Portulaca oleracea L.	QNA-2061	Osman, A. K & Mousa, M.A.	23/5/2024
Pseudognaphalium luteoalbum (L.) Hilliard & B.L.Burtt	QNA-2062	Osman, A. K & Mousa, M.A.	23/5/2024
Psidium guajava L.	QNA-2063	Osman, A. K & Mousa, M.A.	23/5/2024
Raphanus raphanistrum subsp. *sativus* (L.) Schmalh.	QNA-2064	Osman, A. K & Mousa, M.A.	23/5/2024
Ricinus communis L.	QNA-2065	Osman, A. K & Mousa, M.A.	23/5/2024
Rumex dentatus L.	QNA-2066	Osman, A. K & Mousa, M.A.	23/5/2024
Rumex spinosus L.	QNA-2098	Osman, A. K & Mousa, M.A.	13/8/2024
Salix mucronata Thunb.	QNA-2067	Osman, A. K & Mousa, M.A.	23/5/2024
Sesamum indicum L.	QNA-2068	Osman, A. K & Mousa, M.A.	23/5/2024
Sesbania sesban (L.) Merr.	QNA-2069	Osman, A. K & Mousa, M.A.	23/5/2024
Setaria verticillata (L.) P.Beauv.	QNA-2070	Osman, A. K & Mousa, M.A.	23/5/2024
Sida alba L.	QNA-2099	Osman, A. K & Mousa, M.A.	13/8/2024
Solanum lycopersicum L.	QNA-2071	Osman, A. K & Mousa, M.A.	23/5/2024
Solanum nigrum L.	QNA-2072	Osman, A. K & Mousa, M.A.	23/5/2024
Sonchus oleraceus L.	QNA-2073	Osman, A. K & Mousa, M.A.	23/5/2024
Sorghum bicolor (L.) Moench	QNA-2074	Osman, A. K & Mousa, M.A.	23/5/2024

Sorghum virgatum (Hack.) Stapf	QNA-2075	Osman, A. K & Mousa, M.A.	23/5/2024
Symphyotrichum squamatum (Spreng.) G.L.Nesom	QNA-2100	Osman, A. K & Mousa, M.A.	13/8/2024
Tamarix nilotica (Ehrenb.) Bunge	QNA-2076	Osman, A. K & Mousa, M.A.	23/5/2024
Trianthema portulacastrum L.	QNA-2077	Osman, A. K & Mousa, M.A.	23/5/2024
Tribulus terrestris L.	QNA-2078	Osman, A. K & Mousa, M.A.	23/5/2024
Trifolium alexandrinum L.	QNA-2079	Osman, A. K & Mousa, M.A.	23/5/2024
Trifolium resupinatum L.	QNA-2080	Osman, A. K & Mousa, M.A.	23/5/2024
Trigonella anguina Delile	QNA-2081	Osman, A. K & Mousa, M.A.	23/5/2024
Triticum aestivum L.	QNA-2082	Osman, A. K & Mousa, M.A.	23/5/2024
Typha domingensis Pers.	QNA-2083	Osman, A. K & Mousa, M.A.	23/5/2024
Urochloa ramosa (L.) T.Q.Nguyen	QNA-2101	Osman, A. K & Mousa, M.A.	13/8/2024
Urochloa reptans (L.) Stapf	QNA-2084	Osman, A. K & Mousa, M.A.	23/5/2024
Urospermum picroides (L.) Scop. ex F.W.Schmidt	QNA-2085	Osman, A. K & Mousa, M.A.	23/5/2024
Vachellia nilotica (L.) P.J.H.Hurter & Mabb.	QNA-2086	Osman, A. K & Mousa, M.A.	23/5/2024
Vicia faba L.	QNA-2110	Osman, A. K & Mousa, M.A.	15/2/2025
Withania somnifera (L.) Dunal	QNA-2087	Osman, A. K & Mousa, M.A.	23/5/2024
Xanthium strumarium L.	QNA-2088	Osman, A. K & Mousa, M.A.	23/5/2024
Zea mays L.	QNA-2089	Osman, A. K & Mousa, M.A.	23/5/2024
Ziziphus spina-christi (L.) Desf.	QNA-2090	Osman, A. K & Mousa, M.A.	23/5/2024

Table S2. Simple linear correlation coefficient (r) between the estimated soil variables and the DCA axes 1 and 2, *$P < 0.05$, **$P < 0.01$.

Soil variable	DCA axis	
	**Axis 1**	**Axis 2**
Cl^-	**0.84****	0.20
SO_4^{2-}	0.47	-0.20
NO_3^-	0.01	-0.37
K^+	**0.71****	0.17
Na^+	**0.83****	0.14
Ca^{2+}	0.39	-0.01
Mg^{2+}	0.13	-0.05
pH	0.36	-0.02
TDS	**0.70****	0.13
EC	**0.71****	0.12
Organic matter	**-0.88****	-0.33
$CaCO_3$	0.36	-0.34
Field Capacity	**-0.88****	-0.24
Clay	**-0.96****	-0.22
Silt	-0.08	-0.08
Sand	**0.90****	0.23
